# RNA interference (RNAi) applications to the management of fall armyworm, *Spodoptera frugiperda* (Lepidoptera: Noctuidae): Its current trends and future prospects

**DOI:** 10.3389/fmolb.2022.944774

**Published:** 2022-09-07

**Authors:** Megersa Kebede, Tarekegn Fite

**Affiliations:** School of Plant Sciences, College of Agriculture and Environmental Sciences, Haramaya University, Haramaya, Ethiopia

**Keywords:** biological option, gene silencing, RNAi, insect pest management, *Spodoptera frugiperda*

## Abstract

The fall armyworm *Spodoptera frugiperda* (Lepidoptera: Noctuidae) is among the invasive insect pests that damages maize and sorghum, the high-priority crops in newly colonized agro-ecologies, including African contexts. Owing to the increasing infestation of the pest and the limitations of current conventional methods for its management, there is a call for discovering advanced pest management approaches. RNA interference (RNAi) is an emerging molecular tool showing flexible potential for the management of *S. frugiperda*. We conducted a search of the recent application of RNAi literature using Google Scholar and Mendeley to find advanced papers on *S. frugiperda* management using RNAi molecular tools that led to growth inhibition, developmental aberrations, reduced fecundity, and mortality, mainly by disruption of normal biological processes of the pest. Although efforts have been made to accelerate the utility of RNAi, many factors limit the efficiency of RNAi to achieve successful control over *S. frugiperda*. Owing to RNAi’s potential bioactivity and economic and ecological acceptability, continued research efforts should focus on improving its broad applicability, including field conditions. Screening and identification of key target genes should be a priority task to achieve effective and sustainable management of this insect via RNAi. In addition, a clear understanding of the present status of RNAi utilization in *S. frugiperda* management is of paramount importance to improve its efficiency. Therefore, in this review, we highlight the biology of *S. frugiperda* and the RNAi mechanism as a foundation for the molecular management of the pest. Then, we discuss the current knowledge of the RNAi approach in *S. frugiperda* management and the factors affecting the efficiency of RNAi application. Finally, the prospects for RNAi-based insect pest management are highlighted for future research to achieve effective management of *S. frugiperda.*

## Introduction

Fall armyworm, *Spodoptera frugiperda* (J.E. Smith) (Lepidoptera: Noctuidae), a voracious invasive insect pest, is among the key production constraints for several crops, including maize and forage grasses, in areas under invasion, including African countries (Prasanna et al., 2018; Ren et al., 2019; Sisay et al., 2019; Wee et al., 2022). The larvae of *S. frugiperda* cause significant damage or losses to maize and sorghum and threaten food security in Africa (Day et al., 2017; Assefa and Ayalew, 2019; Gichuhi et al., 2020). In response to pest invasion, synthetic insecticides and cultural and mechanical practices are among the suggested management methods (Prasanna et al., 2018; Sisay et al., 2019; Mehlhorn et al., 2021). However, due to pest aggressiveness or superior biological characteristics, none of the aforementioned tactics achieved the required level of control, and the pest continues to cause substantial losses in most of the colonized areas. According to Gichuhi et al. (2020) and Du-Plessis et al. (2020), the management of *S. frugiperda* is challenging due to its biological characteristics, including its ability to survive in various environments and a wide range of hosts. On the other hand, synthetic insecticides are still the major approach for controlling insect pests, yet their use is associated with significant hazards to environmental components and human well-being (Prasanna et al., 2018; Xiao-Shuang et al., 2021). Therefore, the investigation of advanced approaches based on molecular systems is mandatory to achieve effective and sustainable management options against *S. frugiperda*.

Among emerging molecular technologies, RNA interference (RNAi) is a potential biological tool to control target insect species (Olawale et al., 2019; Bai-Zhong et al., 2020; Gurusamy et al., 2020a; Gregoriou et al., 2021; Mehlhorn et al., 2021; Xiao-Shuang et al., 2021). RNAi is a mechanism of gene instruction and an antiviral defense system in a cell, resulting in the sequence-specific degradation of messenger RNA (mRNAs) either by directing inhibitory chromatin modifications or by decreasing the stability or translation potential of the targeted mRNA (Andrew et al., 2001; Liu et al., 2020; Mehlhorn et al., 2021). Double-stranded RNA (dsRNA) generated from an endogenous genomic locus or a foreign source, such as a transgene or virus, is responsible for gene silencing (Liu et al., 2020; Hernández-Soto and Chacón-Cerdas, 2021). The RNAi machinery possesses vital gene “core genes”, which are crucial for its efficiency and strong response in gene silencing (Dowling et al., 2016; Davis-Vogel et al., 2018).

RNAi-based technology shows pronounced potential for use in agriculture, particularly for the management of destructive insect pests, such as *S. frugiperda* (Davis-Vogel et al., 2018; Bai-Zhong et al., 2020; Gurusamy et al., 2020b; Mehlhorn et al., 2021; Wan et al., 2021). RNAi application in Lepidoptera was first reported in 2001 (Bettencourt et al., 2002). Reports have indicated that different insects have shown a wide range of sensitivity to environmentally introduced RNAs. On the other hand, emphasis has been placed on developing dsRNA application strategies for insect management under field conditions through genetically modified organisms (GMOs) in which transgenic plants produce a dsRNA that silences a target insect gene following ingestion and direct application of dsRNA onto plants (Tabashnik and Carrière, 2017; Rosa et al., 2018; Ren et al., 2019; Shuo et al., 2020; DeSchutter et al., 2021). Recently, Rodriguez et al. (2021) demonstrated a sprayable dsRNA bioinsecticide with a novel mode of action that activates the RNAi pathway in *Leptinotarsa decemlineata*. Consequently, there was a sufficient level of efficacy of the sprayable dsRNA product over this insect under both field and laboratory conditions. Insecticidal gene silencing using RNAi encompasses a posttranscriptional mechanism with high potential for target insect pest control (Dias et al., 2020). In other words, field trials are confirming the power of spray-induced gene silencing (SIGS) based bioinsecticides and subsequently promoting their transition to the market, although further improvement is needed to boost the field efficacy of SIGS and sustain RNAi technology (R odriguez et al., 2021; DeSchutter et al., 2021). Currently, promising results in gene lockdown or silencing with a recognized side effect in normal biological activities are being reported in *S. frugiperda* (Rodríguez-Cabrera et al., 2010; Vatanparast and Park, 2022), signifying its potential as a component of pest management. However, the sustainability of these technologies is threatened by increased field resistance in *S. frugiperda* (Tabashnik, and Carriere, 2017; Wang et al., 2017). In lepidopterans, in addition to their refractory nature, several factors, such as the RNAi machinery, dose or concentration of the dsRNA, insect life stage and tissue used, dsRNA molecule delivered, enzymes/double-stranded ribonucleases (dsRNases); duration of exposure, and measurement points, limit the efficiency of insecticidal RNAi (Ruo-Bing et al., 2018; Peng et al., 2019; Rodríguez-de la Noval et al., 2019; Liu et al., 2020; Chen et al., 2021). Studies on RNAi present both positive implications and the limitations associated with this emerging tool, providing a range of possibilities to improve RNAi efficiency and realize its utilization as a potential ecological approach for the management of *S. frugiperda*.

In general, understanding differences in insect responses to RNAi is central to the development and proper implementation of RNAi-based insect pest management. Promisingly, different RNAi-based research findings have been reported for the management of *S. frugiperda*, an invasive insect pest. However, the present knowledge of this technology is not well evaluated with due emphasis on establishing research gaps for further work. On the other hand, searching and evaluating the current information is necessary to obtain knowledge and a better understanding of the present achievement on this pest and prospects to improve the RNAi mechanism and/or to utilize the available technologies. Hence, in this review, the biology of *S. frugiperda* and the RNAi mechanism are emphasized as a basis for the molecular management of the pest. Then, we discuss the current knowledge of the RNAi approach in *S. frugiperda* management and summarize the factors influencing the success of RNAi application. Finally, the potential prospects of RNAi-based technologies are identified for future research to achieve effective management against the pest.

## Methods

A literature search was conducted using Google Scholar and Mendeley (Mendeley-Desktop-1.19.8) with several quest terms. The databases were used because they contained research articles that were available in full text and had undergone peer-review by scientists with advanced search functions. The search terms included the following: (*“S. frugiperda”* and “biology”), (“RNAi mechanism and lepidopterans”), (“*S. frugiperda*” and “biological control”), (“*S. frugiperda*” and “RNAi application”), (“*S. frugiperda*” and “gene silencing”), (“*S. frugiperda*” and “RNAi effects”), (“*S. frugiperda*” and “RNAi delivery methods”), (“*S. frugiperda*” and “RNAi inefficiency”) and (“*S. frugiperda*” and “RNAi prospects”) was used to generate a databank of advanced papers or publications that assess RNAi application efforts in a *S. frugiperda* insect pest management context. The search was limited to the most recent publications that were written in English. Studies that used RNAi-mediated strategies for fall armyworm control, and assessed its effects on biology, physiology and/or behavior were emphasized. Relevant information was extracted from all articles that resulted from the literature search and screening references therein. Accordingly, RNAi application achievements, including target genes, delivery methods, target stage and effects on biological processes (Table 1) and the main factors influencing RNAi-based *S. frugiperda* management (Table 2), and future lines of research are summarized in this review.

**TABLE 1:** Effects of RNAi applications for the management of *S. frugiperda*.

Target gene	Mode of delivery	Target stage	Observed biological consequences in insect	References
Chitinase (*Sf-CHI*)*,* Chitin synthase B (*Sf-CHSB*)*,* Sugar transporter SWEET1 (*Sf-ST*)*,* and Hemolin (*SfHEM*)	Feeding bacteria expressing dsRNAs	larva	Induced silencing (reduction of genes expression) and resulted in significant negative effects on growth and survival. Reductions in expression levels were *Sf*-CHI (66.0–67.9%), *Sf* CHSB (35.0–36.9%), *Sf*-HEM (14.0–16.5%) and *Sf*-ST (15.0–17.5)	Ren et al. (2019); Xiao-Shuang et al. (2021); Mohammad and Youngjin (2022)
*dsCHI* and *dsCHSB*	Injecting of dsRNAs	pupae	Gene silencing and induced a significant increase in wing malformation in adults	Silva et al. (2017); Xiao-Shuang et al. (2021)
Hemolin (*dsHEM*)	Injecting of dsRNAs	adult	Lower mating, fecundity, and egg hatching eggs. Deflation or transparency in eggs, with death, showing abnormal embryonic development	Xiao-Shuang et al. (2021)
Allatostatin (*AS*) and allatotropin (*AT*)	Injections of dsRNA	adult	Gene silencing and reduction of fecundity by approximately 40%	Griebler et al. (2008); Sláma (2013); Baum and Roberts (2014)
Allatostatin (*AS*) and allatotropin (*AT*)	Injections of dsRNA	larva	An increase in larval time to pupation or stunted the larval growth	Griebler et al. (2008); Sláma (2013)
*CeSid1*	Ovarian cells (*Sf9*) and midgut cells (*Sf17*)	adult	Enhanced RNAi efficiency in ovarian *Sf9* cells (reduced accumulation of dsRNA), but not in midgut *Sf17* cells	Rodríguez-de la Noval et al. (2019); Chen et al. (2021)
Peritrophin (*SfPER*)	treated with dsRNA	larva	Reduction of gene expression in the larval midgut (Silencing)	Rodríguez-de la Noval et al. (2019)
peritrophin (*SfPER*)	treated with dsRNA	pupae	Reduction of pupae weight and delaying in adult emergence	Silva et al. (2017); Rodríguez-de la Noval et al. (2019)
peritrophin (*SfPER*)	treated with dsRNA	adult	Inhibited the adult growth performance	Silva et al. (2017); Rodríguez-de la Noval et al. (2019)
*GusA*	treated with dsRNA	larva	100% growth in inbition in larva	Rodríguez-de la Noval et al. (2019)
Vacuolar protein sorting associating protein 4 (*VPS4*) gene	Nanoparticle and siRNA delivered using a nebulizer compressor	larva	PLGA nanoparticles provided a significant decrease in gene expression, while PFC and Chitosan were a slight decrease	Li and Blissard (2012); Vélez and Fishilevich, 2018; Ana et al. (2020); Gurusamy et al. (2020a)
Glycerol biosynthesis gene or glycerol-3-phosphate dehydrogenase (*Sf-GPDH*)	Injection of dsRNAs	larva	Downregulation of gene at the mRNA level, significant decreases with incubation time, a significantly reduced Glycerol amount, reduced cold-tolerance and increased mortality	Park and Kim (2013); Shetty et al. (2019); Xiao-Shuang et al. (2021); Mohammad and Youngjin (2022)
Glycerol biosynthesis gene (*Sf-GK1*)	Injection of dsRNAs	larva	Downregulation of gene (reduced transcription), significantly decreased incubation time, a significantly suppressed glycerol amount, a reduction in survival rate	Park and Kim (2013); Shetty et al. (2019); Mohammad and Youngjin (2022)
Glycerol biosynthesis gene (*Sf-GK2*)	Injection of dsRNAs	larva	Downregulation of gene, significant decreases with incubation time, a significantly suppressed glycerol levels of more than seven times (6.08 mM) and increased the mortality	Park and Kim (2013); Shetty et al. (2019); Mohammad and Youngjin (2022)
Cytochrome P450 (*CYP6BF1v4*) and trypsin protein (*SfT6*) (*Cry1Ca1*)	Droplet feeding	larva	Successful gene silencing was achieved and increased susceptibility to bt toxin	Griebler et al. (2008); Rodríguez-Cabrera et al. (2010)

**TABLE 2:** Some of the factors influencing the efficiency of RNAi application in *S. frugiperda* management.

Basic factors	Supposed driving forces for observed effects or changes	References
Target genes	- Variable RNAi effects and/or efficiency (expression level) among target genes due to the identity (unique nature) of each gene. - Each gene requires specific action to be silenced, thus vigilant selection of the target gene is needed for gene function study. - In lepidopterans, the genes expressed in epidermal tissues are relatively challenging to be silenced	Terenius et al. (2011); Yang et al. (2019); Xiao-Shuang et al. (2021); Chen et al. (2021); Mohammad and Youngjin (2022)
Enzymes (nucleases/REases)	- The quantity or content of nucleases/REases or dsRNases, the nucleic acid degrading enzymes in lepidopterans fluids (saliva, gut lumen, hemolymph) highly determines the dsRNA stability/degradation, and it clarifies why they are refractory to RNAi. - Affect RNAi efficiency due to dsRNA catabolism, especially when administered orally	Terenius et al. (2011); Li and Blissard (2012); Baum and Roberts (2014); Christiaens et al. (2014); Davis-Vogel et al. (2018); Guan et al. (2018); Peng et al., 2018; Ruo-Bing et al. (2018); Rodríguez-de la Noval et al. (2019); Chen et al. (2021)
A nuclease specific gene (*up56*)	- A gene, *up56*, which is upregulated in reaction to dsRNA is responsible for RNAi inefficiency or lepidopterans refractory. - It is a specific gene that encodes nucleases (REases) that contribute to RNAi insensitivity. - Knockdown of *up56* contribute to RNAi efficiency	Tabashnik et al. (2013); Davis-Vogel et al. (2018); Ruo-Bing et al. (2018); Chen et al. (2021)
Delivery methods of dsRNA/siRNA - Diet/feeding -Injection -Recombinant bacteria or ectopic expression of *CeSid1* -Nanoparticles (PLGA, PFC and Chitosan)- Pheromone bait traps	- RNAi efficiency and effects vary among dsRNA delivery methods because each method and/or technique has its own potential of precision/effectiveness to provide (or in delivering dsRNA) under the same or different conditions. - Facilitate or improve the uptake of dsRNA/siRNA - Affect insect feeding behavior - Protect or reduce the dsRNA degradation in the environment and insect	Mon et al. (2012); Xu et al. (2013); Baum and Roberts (2014); Joga et al. (2016); Wu et al. (2016); Ali et al. (2017); Ana et al. (2020); Chen et al. (2021); Xiao-Shuang et al. (2021)
The insect status-Physiological status- Insect growth stage-Sex -Tissues	Variable RNAi efficiency as a consequence of different gene expression levels, is mainly due to the nature of contributing insect status and/or corresponding differences	Ulvila et al. (2006); Ren et al., 2014; Davis-Vogel et al. (2018); Rodríguez-de la Noval et al. (2019); Cooper et al. (2019); Chen et al. (2021); Mohammad and Youngjin (2022)
The insect tissue (gut) - gut cells - gut pH	- The gut, mainly mid-gut contains nucleic acid degrading enzymes that are capable of degrading dsRNA, and consequently affect RNAi. - A strong alkaline pH found in lepidopterans gut can offer a hostile condition for dsRNA that contribute to their recalcitrance to RNAi	Yoon et al. (2017); Davis-Vogel et al. (2018); Yang et al. (2019); Chen et al. (2021); Chen et al. (2021)
dsRNA uptake and length	- The uptake of dsRNA by the cell and the involving mechanisms are critical factors in determining the effectiveness of RNAi. - Differences in the core RNAi machinery are the main causes of variation in dsRNA uptake in addition to its degradation by nucleases prior to taken-up or processed which leads to variations in gene expression - Length of dsRNA influences RNAi mechanisms or efficiency there by ensuring the molecular uptake	Huvenne and Smagghe (2010); Davis-Vogel et al. (2018); Maktura et al. (2021)
dsRNA design and concentration	- dsRNA design defines the specific target gene, however, nontarget effects can occur due to siRNAs sequence similarity with nontarget genes and this improved resistance in lepidopterans, especially *S. frugiperda* - Ideal dsRNA amount is needed to achieve sufficient silencing. However, sensitivity to dsRNA is mostly based on the target gene	Zhu et al. (2012); Uryu et al. (2013)
Encapsulation System poly-[N-(3-guanidinopropyl) methacrylamide] (pGPMA)/dsRNA interpolyelectrolyte Nanocomplex	-Increased internalization and protection of dsRNA in insect cells, decreasing the accumulation of target mRNA due to the knockdown of genes related to vital functions such as nutrient absorption (*sfVATPase*), intracellular transport (*sfKIF*), and cell division (*sfCDC27*)	Li and Blissard (2012); Baum and Roberts (2014); Parsons et al. (2018); Vélez and Fishilevich, 2018; Ana et al. (2020)
Encapsulation System Chitosan/dsRNA polyplex nanoparticles	Improves RNAi efficiency through the protection of dsRNA from degradation by intracellular and intercellular RNases. It also reduces the accumulation of dsRNA in the endosome while favoring its transport to the cytoplasm, where the formation of siRNAs is promoted, producing knockdown of apoptosis-related genes	Vélez and Fishilevich (2018); Ana et al. (2020); Gurusamy et al. (2020a)
Encapsulation System *CeSid1* expression/dsRNA.in late endosomes	*CeSid1* protein facilitates the uptake of dsRNA in the insect’s gut and achieved effective gene silencing in *Sf9* cells. It reduces the accumulation of dsRNA in late endosomes and facilities the processing of dsRNA to siRNA in *Sf9* cells. The percentage of dsRNA localized with late endosomes was reduced by ∼65% in *Sf9_Luc_CeSid-1* stable cells compared to those in *Sf9*_*Lucstable* cells	Christiaens et al. (2018b); Ren et al. (2019); Xiao-Shuang et al. (2021); Chen et al. (2021)

### Biology of S. frugiperda

A better understanding of the biology of *S. frugiperda*, an invasive insect pest, is a foundation for its management in noninvasive ways, particularly through molecular options. *S. frugiperda* is native to America and remained a pest on the continent until its out-break was described in Nigeria, West Africa, in 2016 (Cock et al., 2017; Goergen et al., 2017; Nagoshi et al., 2017; Otim et al., 2018). Presently, its occurrence has been proven on five continents, including Africa (Michael et al., 2021). As an invasive pest*, S. frugiperda* has a pronounced capacity to occupy wide ecological zones in a short period (Johnson, 1987; Du -Plessis et al., 2020). The rapid dispersal of *S. frugiperda* is associated with its biological features, notably high reproductive rates, strong migration capacity, and extensive host range (Gichuhi et al., 2020). Interestingly, although it is polyphagous, *S. frugiperda* prefers to feed on maize, causing substantial yield penalties in invasive ranges, particularly in African countries (Day et al., 2017; Michael et al., 2021).

The insect undergoes holometabolosis, and its life cycle includes eggs (2–3 days), larvae (13–14 days), pupae (7–8 days) and adults (7–21 days) (Jing-Wan et al., 2021; Michael et al., 2021). *S. frugiperda* has a generation time of approximately 30–40 days during the warm summer months (daily temperature of ∼28°C) and approximately 55 days at cooler temperatures (Prasanna et al., 2018; Sharanabasappa et al., 2018). The individual adult female can lay 1,500–2000 eggs in her life (Johnson, 1987). The eggs are laid on leaves in batches containing 100–200 eggs (Johnson, 1987; FAO, 2019). The eggs hatch in two to 3 days during summer (20–30°C) (Capinera and Smith, 1999; Sharanabasappa et al., 2018). There are six larval instars with a development duration of two to 3 weeks, with the last instar being the most destructive (causing 70% of *S. frugiperda* damage) (Assefa and Ayalew, 2019). The pest cannot enter diapause (Johnson, 1987); hence, the number of generations produced in invaded areas is determined by the suitability of environmental situations, mainly temperatures coupled with the host plants (Prasanna et al., 2018; Du -Plessis et al., 2020); for instance, in tropical zones, it can have up to eight generations in 1 year in maize fields (Busato et al., 2005).

At present, two strains of *S. frugiperda*, namely, the “corn” (C strain) and the “rice” (R strain), are known in both the native and invasive ranges (Nagoshi et al., 2019). Accordingly, the corn strain damages corn/maize, sorghum and cotton, but the rice strain damages rice, silk grass and forage grasses. Despite their morphological similarity, however, the two strains differ in genetics, physiology, and behavioral features, such as mating and insecticidal resistance (Pashley, 1988; Nagoshi et al., 2018). *S. frugiperda* is known as a super pest due to its extensive host range (more than 350 plant species) from 76 plant families (Montezano et al., 2018), its inherent capacity to live in extensive habitats (Jing-Wan et al., 2021), and its remarkable mobility, high fecundity, quick resistance development to insecticidal treatments or external bodies and gluttonous characteristics (Huang et al., 2019; Gichuhi et al., 2020; Gui et al., 2020; Wee et al., 2022). Notably, the distinct inherited biological features of *S. frugiperda* enhance its invasiveness (Jing-Wan et al., 2021; Wee et al., 2022). In other words, genome research has greatly assisted in understanding the invasive mechanisms of alien species (Wan et al., 2019); mainly, genomic data mining has improved our understanding of the biological and behavioral characteristics of *S. frugiperda* contributing to invasiveness (Gimenez et al., 2020; Gui et al., 2020; Xiao et al., 2020; Zhang et al., 2020; Jing-Wan et al., 2021) and is suggested to accelerate the ecological management of the pest both in native and newly invaded ranges.

Several proteins, including enzymes, control every biological function in insects, including the respiratory, digestive, muscular, reproductive, circulatory, neurological, and endocrine systems. For instance, the important insect neuropeptides allatotropin and allatostatin prevent the synthesis of juvenile hormones (Teal, 2002; Griebler et al., 2008; Abdel-latief and Hoffmann, 2014). Optimizing RNAi bioassays for insects requires an understanding of their feeding habits. Examples of artificial feeding bioassays for insects with piercing-sucking mouthparts are employed frequently for RNAi, which is a crucial factor to take into account when creating an efficient RNAi control approach against plant sap feeders (Andrade and Hunter 2016).

Furthermore, continuous genomic functional studies should be emphasized to identify the key genes for its susceptibility to molecular management of *S. frugiperda*, mainly RNAi interference.

### RNAi mechanism

RNAi is a gene silencing mechanism by target gene degradation of mRNA triggered by dsRNA (Andrew et al., 2001; Hernández-Soto, and Chacón-Cerdas, 2021). The RNAi strategy was detected in 1998 when the nematode *Caenorhabditis elegans* was exposed to dsRNA injection-induced gene silencing (Fire et al., 1998). Currently, there are reports on promising achievements in RNAi-based insect pest management. Moreover, understanding the mechanism of RNAi is key to utilizing this technology against target insect species. In general, the RNAi mechanism involves two main events: first, exogenously delivered dsRNAs are internalized and converted by the cellular RNAi machinery into small interfering RNAs (siRNAs), which then activate the degradation of complementary endogenous mRNAs, leading to gene silencing (Andrew et al., 2001; Tomari and Zamore, 2005; Zotti et al., 2018; Maktura et al., 2021).

**FIGURE 1:**
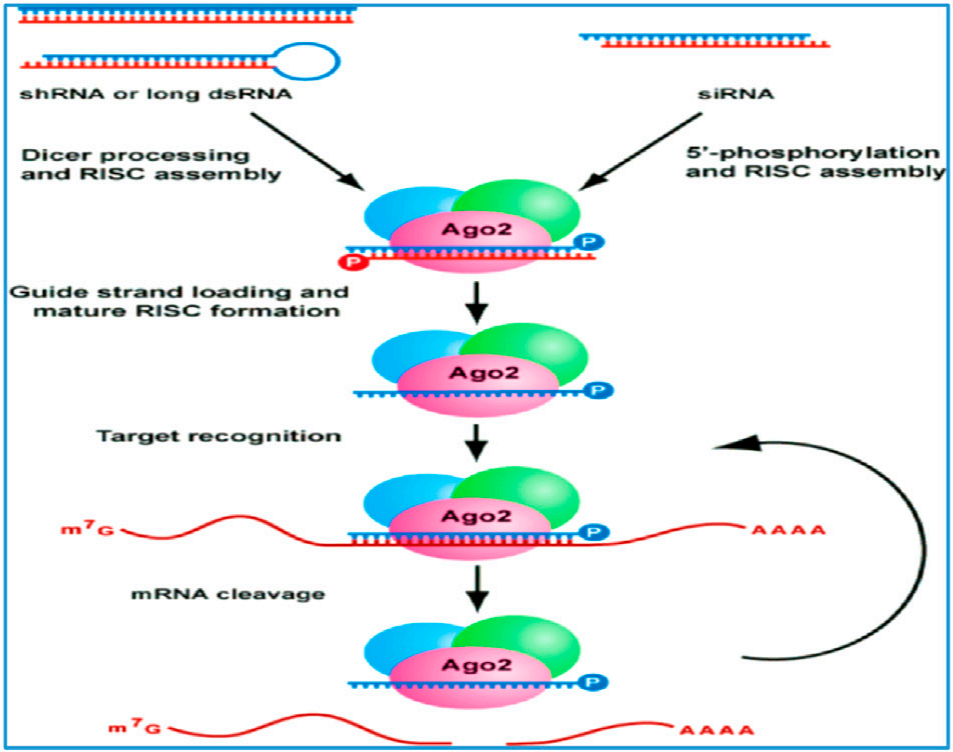
RNA-mediated gene silencing pathways, adapted from Andrew et al. (2001)

Briefly, when a dsRNA molecule targeting a specific gene enters the cell, a ribonuclease III enzyme, Dicer-2 (Dcr-2), recognizes it and fragments it into 21–24 bp molecules, siRNA. A dsRNA-binding protein named R2D2 connects Dcr-2-bound siRNA to the RNA-induced silencing complex (RISC) formed by the Argonaute-2 (Ago-2) protein and other proteins (Ye et al., 2019). Ago-2 unwinds the siRNA duplex and keeps the guide strand connected to RISC. Then, this strand becomes a guide for RISC to bind specifically to target mRNA molecules, and Ago-2 severs the complementary mRNA, resulting in posttranscriptional gene silencing (Joga et al., 2016), as illustrated in Figure 1 below. Although the basic RNAi mechanism exists in all insects, there are important differences in the machinery compounds with respect to their sensitivity to systemic RNAi, such as modifications (Davis-Vogel et al., 2018; Xiao-Shuang et al., 2021), duplications or loss of RNAi-related genes (Dowling et al., 2016), which may explain differences in RNAi effects between insect orders or species. In insects, three RNAi pathways have been detected, namely, the dsRNA/siRNA-mediated siRNA pathway, miRNA-mediated miRNA pathway and pian-interacting RNA (piRNA)-mediated piRNA pathway (Zhou et al., 2008; Han et al., 2015; Zhu and Palli, 2020; Maktura et al., 2021). In addition, further inquiry for a better understanding of RNAi mechanisms or pathways is necessary to enhance its application and/or efficiency in devising sustainable insect pest management.

### RNAi applications IN *S. frugiperda*

#### Growth and development

**FIGURE 2:**
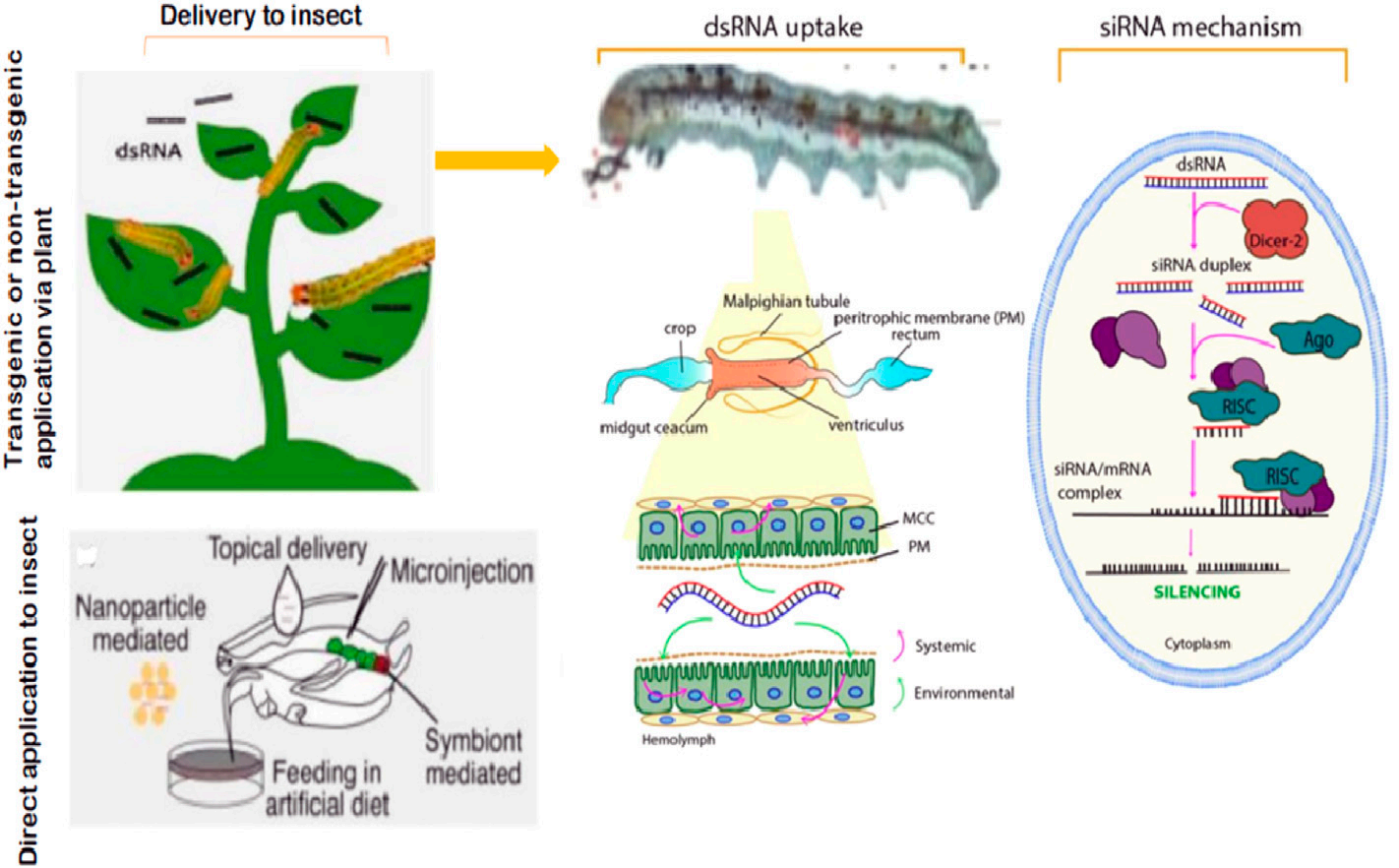
Application strategies and the basic cellular mechanism of RNAi-mediated plant protection from a lepidopteran pest control standpoint.

Most studies using RNAi in *S. frugiperda* are focused on growth regulating hormones or involving genes to inhibit insect growth or survival. Consequently, remarkable effects of RNAi have been reported to disrupt normal biological processes, particularly growth and development in *S. frugiperda* (Griebler et al., 2008; Xiao-Shuang et al., 2021; Chen et al., 2021; Mohammad and Youngjin (2022)), and some of these effects are summarized in Table 1. The application strategies, including the cellular mechanism of RNAi-mediated gene silencing to combat the insect pest, are presented in Figure 2. Feeding or injection of *S. frugiperda* with dsRNA provided efficient gene transcripts in the midgut and brain tissue, posing significant growth inhibition (Griebler et al., 2008; Rodríguez-Cabrera et al., 2010). Griebler et al. (2008) demonstrated silencing of allatostatin and allatotropin production in adult females of *S. frugiperda*. Additionally, they confirmed that silencing allatostatin led to an increase in peak larval weight while silencing allatotropin resulted in retarded growth and weight, in treated larvae. Hassanien et al. (2014) also indicated that *Spofr-AT* gene silencing acts as a true allatotropin in larvae and adults of both sexes of *S. frugiperda.* Likewise, the injection of *S. frugiperda* adults and larvae with dsRNA silences the production of allatoregulating neuropeptides (Griebler et al., 2008). Efficient silencing by dsRNA by feeding was highly enhanced when larvae were used immediately after the molt and in addition were subjected to starvation for 24 h (Rodríguez-Cabrera et al., 2010), indicating that the degradation of dsRNA in the midgut of larvae was greatly decreased due to starvation and could be an important factor for the increased sensitivity to dsRNA. Juvenile hormone is a multipurpose hormone that contributes to young growth regulation, reproduction, metabolism, and diapause (Sláma, 2013). Allatostatin and allatotropin are produced by the brains of insects to depress or stimulate corpus allatum production of juvenile hormones, respectively (Sláma, 2013). Hence, it could be concluded that improvement in the application of RNAi targeting juvenile hormones (allatostatin and allatotropin) or involving genes could contribute to the effective management of the pest.

On the other hand, following the increasing trends in the improvement of dsRNA/siRNA delivery methods, the efficiency of RNAi in gene silencing has been enhanced, providing a remarkable adverse effect on insect growth and development. Ectopic expression of *CeSid1* enabling the passive uptake of dsRNA from the culture medium was first confirmed in *Drosophila melanogaster S2* cells (Feinberg and Hunter, 2003), and effective gene silencing was achieved in *S. frugiperda Sf9* cells expressing *CeSid1* (Mon et al., 2012; Xu et al., 2013; Xiao-Shuang et al., 2021). Feeding *S. frugiperda* with bacteria expressing dsRNA (Xiao-Shuang et al., 2021) and systemic RNAi defective protein 1 (*CeSid1*) negatively affected the growth, development and survival of the insect (Chen et al., 2021). According to Xiao-Shuang et al. (2021), feeding larvae with bacteria expressing dsRNAs of target genes or injecting pupae and adults with bacterially synthesized dsRNA induced silencing of target genes (*Sf-CHI, Sf-CHSB, Sf-ST* and *Sf-HEM*) and resulted in considerable adverse effects on the development and survival of *S. frugiperda.* A significant decrease in larval body weight, with reduced rates of 12.96–28.10%, was noted due to the knockdown of these genes (Xiao-Shuang et al., 2021). Likewise, lower survival rates and prolonged developmental duration were observed following RNAi targeting of genes involved in chitin production in *S. frugiperda* (Pesch et al., 2016; Zhao et al., 2018). The growth and development of insects are firmly dependent on the precise regulation of chitin synthesis and digestion (Pesch et al., 2016; Zhao et al., 2018) and are responsible for chitin synthesis in the midgut and peritrophic membrane of larvae (Zhang et al., 2012; Yang et al., 2019. Interestingly, injection of pupae with *dsCHI* significantly reduced the enclosion rate of pupae but increased wing malformation in adults (Xiao-Shuang et al., 2021). Chitin is the main substance of insect wings, and the precise regulation of chitin synthesis and digestion may be crucial for the development of wings (Zhang et al., 2012; Pesch et al., 2016; Zhao et al., 2018) and result in adult wing malformation after the knockdown of *Sf-CHI* in *S. frugiperda* (Xiao-Shuang et al., 2021). RNAi targeting *Sf-GK2* and *Sf-GK1* reduces glycerol biosynthesis or content and consequently affects the growth and development of *S. frugiperda* by knocking down these genes (Shetty et al., 2019; Mohammad and Youngjin (2022)). RNAi of glycerol biosynthesis genes (*Sf-GPDH, Sf-GK1* and *Sf-GK2*) increases the mortality of treated larvae of *S. frugiperda* (Mohammad and Youngjin, 2022), which suggests that downregulation of the genes involved in the environmental adaptability of the insect has a paramount role in achieving effective management. In other words, the effect of RNAi on the growth, development and survival of *S. frugiperda* varied based on the target gene, tissue, growth stage, means of dsRNA/siRNA delivery and others (Yang et al., 2019; Xiao-Shuang et al., 2021; Chen et al., 2021; Mohammad and Youngjin, 2022), and some of these factors are presented in Table 1 below. Consequently, further study is needed to identify different factors as well as the involved mechanism of RNAi inefficiency in *S. frugiperda* to progress its effectiveness against the pest*.*

Recent studies have shown that systemic RNAi in *Caenorhabditis elegans* requires systemic RNAi defective protein 1 (*CeSid1*) to achieve downregulation of genes and adversely affect insect growth or survival. Chen et al. (2021) suggested the tissue-specific enhancement of RNAi efficiency by *CeSid1* in *S. frugiperda* for improving RNAi in insects such as those belonging to the order Lepidoptera where RNAi is variable and inefficient. For example, the expression of *CeSid1* enhanced RNAi efficiency in ovarian *Sf9* cells but not in midgut *Sf17* cells in *S. frugiperda*. Interestingly, *CeSid1*-expressing transgenic *S. frugiperda* revealed a 93.3% reduction in gene expression in tissues such as Verson’s glands compared to wild-type. The reduced accumulation of dsRNA in late endosomes and successful processing of dsRNA to siRNA contribute to enhanced RNAi efficiency in *Sf9* cells (Chen et al., 2021). Further examination of *CeSid1* and the mechanism through which this dsRNA transporter improves RNAi efficiency in this insect is necessary. Injection of dsRNA into the abdomen of *Blattella germanica* caused dramatic depletion of essential α-tubulin gene expression in the insect’s midgut and yielded recognized mortality (Lin et al., 2016). Likewise, Laisney et al. (2020) showed that luciferase gene knockdown was more efficient by exposing Sf9 cells to nanoscaled (size <100 nm) compared to microscaled (size >1 µm), providing 58 and 20%, respectively, with a notable side effect on *S. frugiperda* larvae. These studies signify the need for the development of an effective delivery system in addition to selecting potential candidate tissues or cells to achieve a required level of gene silencing via RNAi. On the other hand, since the insect midgut is in close proximity to the site of dsRNA entry, the potential of midgut genes as targets for RNAi has been investigated, including genes involved in peritrophic membrane (*PM*) synthesis providing a recognized reduction in the growth and reproduction of *S. frugiperda* (Rodriguez-Cabrera et al., 2010; Toprak et al., 2013; Kelkenberg et al., 2015; Ni et al., 2017). Rodríguez-de la Noval et al. (2019) and Silva et al. (2017) demonstrated that peritrophin (*SfPER*) is a new *PM CMCMC-*type peritrophin A-like protein from *S. frugiperda*, whose reduction using RNAi decreases pupal weight and adult emergence, two important parameters of insect development and fitness due to alterations in *PM* functions related to digestion and nutrition (Table 1). Furthermore, the findings suggested that *SfPER* could be a potential target for novel pest-control strategies in *S. frugiperda*. It is particularly beneficial to use gene silencing in the field since its effects are independent of additional behavior or supplementary techniques, leading to impaired development and mortality. Once the delivery difficulty is overcome, delays in growth or development will immediately lessen the number of insects and the resulting damages.

#### Reproduction

In lepidopterans, an alteration of the reproductive process is an alternative for designing RNAi-mediated insect control strategies (Bettencourt et al., 2002; Rajan et al., 2013). RNAi targeting of different genes causes downregulation or silencing and consequently results in disruption of reproduction in *S. frugiperda* (Griebler et al., 2008; Shu et al., 2011; Xiao-Shuang et al., 2021). Silencing of allatostatin and allatotropin production in adult females of *S. frugiperda* reduced fecundity by approximately 40% (Griebler et al., 2008). Likewise, a study on the reproductive fitness of *S. frugiperda* showed that female adults injected with Hemolin (*dsHEM*) had a significantly lower mating rate than wild-type males and laid fewer eggs with a lower hatching rate (Xiao-Shuang et al., 2021). Researchers have suggested that *Sf-HEM* has a vital role in the course of egg production, especially disturbing mating behavior or female interest (Ringo, 1996; Wedell, 2005) and embryo development (Xiao-Shuang et al., 2021). However, the mechanism of Hemolin (*Sf-HEM*) in reproduction remains unclear and requires further investigation. Some of the effects of RNAi on the reproduction system of *S. frugiperda* are summarized in Table 1. The heritable RNAi effects on the embryos of the subsequent generation were noticed in lepidopterans following injection of dsRNA into pupae, signifying the entrance of the dsRNA into the gonads of the developing pupae (Bettencourt et al., 2002), and the vitellogenin receptor was also detected as a critical component for binding vitellogenin and transporting it into the oocytes in *Spodopteran* species (Shu et al., 2011; Xiao-Shuang et al., 2021). The successful achievements in gene knockdown or silencing with significant adverse effects on reproduction make RNAi a promising tool against *S. frugiperda.* Recent studies have shown that the fertility and the total number of eggs laid per adult female of *S. frugiperda* were decreased by the particular regulation of *Sf-PBANr* (neuropeptide receptor activating pheromone biosynthesis) by RNA interference (Park and Vatanparast, 2022). Therefore, it is advisable to improve RNAi efficiency by focusing on gene functional studies for the identification of key genes that govern *S. frugiperda* reproduction and ways of delivery to sustain its applicability.

#### Insecticide resistance management via gene pyramiding and novel co-formulants

In lepidopteran species, mostly *S. frugiperda*, RNAi is used as a tool to establish the functional relevance of cytochrome P450 and esterase genes to susceptibility against insecticides (Griebler et al., 2008; Hu et al., 2014) and Aminopeptidase-N (*APN*), cadherin, and ATP-binding cassette (*ABC*) transporter genes in susceptibility to *Bt.* Proteins (Gordon and Waterhouse, 2006; Guo et al., 2015; Wang et al., 2017). The application of RNAi machinery as a novel mode of action to control *S. frugiperda* is a weapon identified to halt the increasing difficulty of pest resistance to insecticides and transgenic crops (Jonathan, 2015). Moreover, most of the RNAi studies provided different levels of gene knockdown rather than complete silencing, and these situations are more of a boost to possibilities than a process available for integrated pest management (IPM) implementation (Griebler et al., 2008; Baum and Roberts, 2014). Therefore, this signifies the potential of RNAi to enhance the susceptibility of lepidopterans to products with verified efficiency when used as a component of IPM, especially in the case of *S. frugiperda,* to halt its superior biological characteristics.

Plant-mediated RNAi by genetically improved crops expressing dsRNA (Figure 2) is one of the most promising pest control strategies (Tabashnik and Carrière, 2017; Ren et al., 2019). Interestingly, RNAi can play an important role in *Bt.* crops to delay resistance to *S. frugiperda*, especially when coupled with other management options to achieve the goal of IPM strategies. According to Ren et al. (2019), stacking multiple RNAi expression cassettes against multiple key targets of the pest will successfully achieve useful agronomic traits for crop protection and production. RNAi attacks on several key genes of the midgut may produce synergistic action against *S. frugiperda*, which is promising for disrupting or blocking the level of resistance alleles. On the other hand, strong synergistic interactions between *Vip3* and *Cry proteins,* including *Cry1Ab, Cry2Ab, Cry1Ca* and *Cry1Ea,* have been observed in *S. frugiperda* (Tabashnik and Carrière, 2017). Moreover, combining *Bt.* gene pyramiding, in which diverse Bt toxins can fix to various receptors of pests, with plant-mediated RNAi can targeting multiple key genes for the development, detoxification, digestion and defense of pests (Ren et al., 2019).

Advances in the formulation and delivery of dsRNA play a prominent role in enhancing the efficacy of insecticidal RNAi in controlling lepidopteran insects, including *S. frugiperda,* by limiting their superior or resistance features. The use of potential coformulants for the successful delivery of dsRNA could contribute to improving the effectiveness of gene silencing by designated RNAi. Recently, most studies explained the increments in target cellular uptake following the utilization of lectins and peptides, protein-based carriers (Shen et al., 2017; Parsons et al., 2018; DeSchutter et al., 2021; Martinez et al., 2021). Martinez et al. (2021) reported the high capability of *Galanthus nivalis* lectin (GNAF) fusion protein for complexing with dsRNA and significantly increasing the rate of dsRNA uptake in lepidopteran midgut *CF1* cells. Accordingly, feeding of GNAF: dsV-ATPase by targeting the essential gene V-ATPase-A increased mortality up to 48% in the GNAF-dsRNA treatment compared to only 8.3% and 6.6% in the control treatments with the naked dsRNA and the GNAF, respectively, and impaired development in *S. exigua* larvae (Martinez et al., 2021). Although the complexation mechanism of GNAF in protecting dsRNA for RNAi efficacy remains unclear, lectin-based carriers could be added to the list of carriers that offer promising prospects for use in the lepidopteran insect pest management context (Shen et al., 2017; Martinez et al., 2021). In other words, further investigation is suggested to clarify the exact uptake mechanism of these lectins into insect midgut cells in addition to validating the evidence for effective management of *S. frugiperda*. The conjugation of RNA to cell membrane penetrating peptides has been reported to improve the stability of dsRNA, protect dsRNA against nucleolytic degradation and facilitate efficient internalization into the target cells of lepidopteran larvae, mainly the midgut epithelium (DeSchutter et al., 2021). Interestingly, Parsons *et al.* (2018) proposed the use of synthetic polymer mimics of cell-penetrating peptides to allow efficient internalization into *S. frugiperda* midgut epithelial cells. Generally, silencing the key genes associated with various functions of gut cells in *S. frugiperda* through RNAi is the core for pyramiding and silencing several genes and thus could be an effective way of managing this pest. Moreover, the usage of potential candidate co-formulants of dsRNA for efficient delivery as well as deployment of an integrated tactic could provide several opportunities for the investigation of successful and reproducible environmental RNAi in *S. frugiperda*.

### Factors influencing RNAi efficiency

**FIGURE 3:**
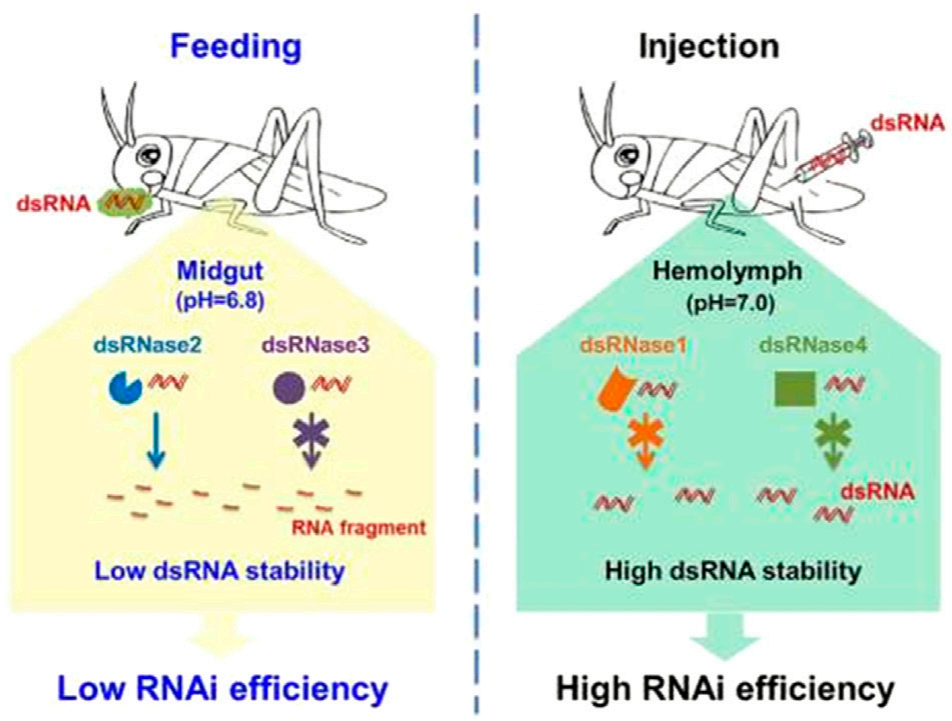
Variable RNAi response of insects associated with feeding and injection as dsRNA delivery methods, adapted from Song et al. (2018)

Despite the prominent potential of RNAi in genomic studies and the management of agriculturally important insect pests (Cruz et al., 2018; Davis-Vogel et al., 2018; Kunte et al., 2020), the instability of RNAi effectiveness among insects limits its application in pest management (Davis-Vogel et al., 2018; Chen et al., 2021). Different factors have been noted to determine the efficiency of RNAi in the management of lepidopteran insect pests, including *S. frugiperda*. An overview of some of the factors affecting RNAi efficiency in *S. frugiperda* is given below (Table 2). RNAi machinery genes, dsRNA uptake and systemic RNAi, delivery methods (Figure 3 presents the case of feeding and injection), RNAi effect variation related to the target gene, dsRNA persistence in the insect body, resistance to dsRNA and differences among insect populations/lineages are the major factors involved in RNAi efficiency (Cooper et al., 2019; Liu et al., 2020; Xiao-Shuang et al., 2021). Hence, validation of RNAi-based management technology for each insect development stage and core machinery gene is mandatory before its usage in large-scale farms to achieve successful management of *S. frugiperda*.

RNAi efficiency in lepidoptera, including *S. frugiperda*, shows high variability, and it is difficult to attain the required level of effectiveness, as in some cases, the increased instability and reduced intracellular transport of dsRNA are challenging factors for RNAi efficacy (Shukla et al., 2016; Peng et al., 2019; Rodríguez-de la Noval et al., 2019; Yang et al., 2019). Similarly, Ruo-Bing et al. (2018) documented a gene called up56, which is upregulated in reaction to dsRNA in lepidoptera. It encodes a nuclease that complements RNAi insensitivity in this group of insects.

By injecting dsRNA into plant vessels or providing it through roots to phytophagous insects (Figure 3), who naturally consume dsRNA through sucking or chewing, dsRNA could be delivered to these insects (Andrade and Hunter, 2016). Furthermore, Hunter et al. (2012) confirmed the translocation of dsRNA supplied exogenously to citrus plants through the vascular system of the plant, which can be taken up by certain xylem/phloem-feeding insect pests of citrus.

While the pH of an insect’s hemolymph ranges from 6.4 to 7.5, the pH of its gut lumen varies greatly (Harrison, 2001). Hindgut pH ranges from neutral to acidic, and crop pH is often acidic (Harrison, 2001). Lepidopterans have extremely alkaline midguts (pH > 8.0), while coleopterans and hemipterans have slightly acidic midguts. Dipterans, hymenopterans, and orthopterans typically have alkaline midguts (Harrison, 2001; Swevers et al., 2013). However, alkaline environments in the guts of dipterans, hymenopterans, and orthopterans may also reduce RNAi efficacy by hydrolyzing dsRNA. The varied alkaline environment of the lepidopteran gut is particularly worrisome for dsRNA stability. Several gut environmental factors influence the effectiveness of oral RNAi in insects by directly impacting dsRNA stability and indirectly influencing the activity of nucleases in the gut (Cooper et al., 2018). This shows that variations in gut pH affect dsRNase activity and may also cause dsRNA hydrolysis, which in turn contributes to low RNAi efficiency (Cooper et al., 2019). In insects with dsRNA stability concerns, additives that change the pH or enzymatic activity in the gut or saliva may also be beneficial for improving RNAi effectiveness.

Similarly, dsRNases clearly define why the RNAi response is limited in Spodopteran species, including *S. frugiperda* (Guan et al., 2018; Peng et al., 2019; Yao et al., 2022). Degradation of dsRNA by dsRNases is thought to contribute to the variability in RNAi efficiency observed among insects (Peng et al., 2019; Wang et al., 2019). The level of dsRNases expression determines the extent of dsRNA degradation and/or RNAi efficiency in *spodopteran* species (Guan et al., 2018; Peng et al., 2019). In other words, the extent of dsRNA expression can be influenced by various factors, including the types of dsRNases, insect growth stage, tissues, target genes, feed, and dsRNA delivery methods (Peng et al., 2019; Yao et al., 2022). For instance, the level of expression of the same or different dsRNases varies across the growth stage or larval instars of *Spodoptera litura*, showing the variable level of dsRNases in response to the changes in the growth stage (larval instars) of insects. Accordingly, the mRNA levels of three midgut-specific dsRNases were amplified during the larval stage, and the maximum dsRNA-degrading action was noticed in third-instar larvae (Peng et al., 2019). Moreover, the authors recognized six genes, namely, dsRNase1, dsRNase2, EndoG, dsRNase3, dsRNase4, and dsRNase5, from *S. litura*. Recent studies proved that multiple dsRNases encoded by the lepidopteran genome could contribute to the lower and inconsistent RNAi efficiency in this insect group in a broad sense (Ruo-Bing et al., 2018; Peng et al., 2019; Yao et al., 2022). Interestingly, Yao et al. (2022) demonstrated the involvement of multiple dsRNases in exogenous dsRNA degradation in *S. frugiperda* larvae. This evidence provides a significant implication in the manipulation of dsRNases and/or influencing factors to reduce dsRNA degradation and increase the RNAi efficiency against *S. frugiperda.* Therefore, further genomic studies should focus examining all aspects of dsRNases, including the mechanism of action in *S. frugiperda,* to lessen the ongoing challenges in dsRNA degradation in the deployment of RNAi. In addition, the success of RNAi in *S. frugiperda* differs among host stage, target gene and tissue or organ as well as the amount of dsRNA (Scott et al., 2013; Ren et al., 2019; Xiao-Shuang et al., 2021) (Tables 2).

Efforts have been made to increase the efficiency of RNAi, and a recent study indicated that chitosan nanoparticles and the Cellfectin II (*CFII*) transfection reagent helped dsRNA escape from endosomes and increased the RNAi response in *S. frugiperda* (Ana et al., 2020; Gurusamy et al., 2020b). The protamine-lipid-dsRNA nanoparticles, according to a more recent study by Dhandapani et al. (2022), increase RNAi efficiency in the target insect species *S. frugiperda* by shielding dsRNA from nuclease degradation and thereby significantly increasing the stability of dsRNA in the organism. Similarly, according to McGraw et al. (2022), clathrin-mediated endocytosis and macropinocytosis account for the majority of cellular uptake, and once inside the midgut, transcytosis is involved in moving branched amphipathic peptide nanocapsules (BAPCs) dsRNA from the lumen to the hemolymph of *S. frugiperda*. RNAi bioinsecticide may not last long in the environment, so extra effort is needed to perform the spraying operation at the right time, which in turn challenges its adoption by growers (Pedigo and Rice, 2009). In other words, the concentration at which the RNAi spray will be effective bears the burden of both cost and field efficacy (Pedigo and Rice, 2009; Baum and Roberts, 2014). In contrast, Baum and Roberts (2014) suggest a tropical application such as nanoparticles to enhance the efficiency of RNAi-based pest management strategies for *S. frugiperda*. Generally, delivery of dsRNA to this insect (Tables 1 and 2) has been documented by different techniques, including injection, feeding on dsRNA synthesized *in vitro* or produced by bacteria or transgenic crops, soaking, or produced in viral and bacterial vectors (Christiaens et al., 2018b), and the effectiveness of RNAi in *S. frugiperda* varies, mainly due to the insect status, delivery method, gene or tissue of interest, and refractoriness to dsRNA (Ruo-Bing et al., 2018; Cooper et al., 2019; Chen et al., 2021; Mohammad and Youngjin, 2022). However, further study is compulsory to identify all the possible limiting factors of the RNAi approach and the underlying mechanisms in this insect, in addition to the assessment of potential safety risks.

### Prospects of RNAi applications in *S. frugiperda*

Lepidopterans are generally refractory to effective gene silencing by RNAi, especially when using oral delivery of dsRNA (Dias et al., 2020), and this is notably associated with the inherited superior biological features in the case of *S. frugiperda,* which boost its adaptation against most of the utilized options, especially when they are applied in a single fashion, recognizing its direct and indirect effects in the case of RNAi as an emerging approach. Despite limiting factors, as an efficient tool in gene function, RNAi enhances susceptibility to insect pests (Xiong et al., 2016; Wang et al., 2017; Mehlhorn et al., 2021) and thus could be considered a promising component in the development of integrated management against *S. frugiperda*. In other words, promising options have been provided to overcome the challenges associated with RNAi applications in lepidopteran *S. frugiperda*. For example, the use of modified or concatemerized dsRNAs has been suggested as a strategy to improve RNAi efficiency (Christiaens et al., 2018a; Sharath et al., 2018; Vélez and Fishilevich, 2018; Ren et al., 2019; Ana et al., 2020; Gurusamy et al., 2020b; Chen et al., 2021; Xiao-Shuang et al., 2021). Presently, many successes in ds/siRNA delivery systems have been proven to contribute to the improvement of RNAi efficiency in *S. frugiperda*, inviting further investigations to achieve a required level of management against the pest. Sprayable dsRNA based bioinsecticide is being reported with successful efficiency under field conditions against *Leptinotarsa decemlineata* (Rodriguez et al., 2021), and this explains great possibilities for use in the case of *S. frugiperda*. Baum and Roberts (2014) alluded to a tropical application for RNAi-based pest management strategies to protect ds/siRNAs from the environment to control *S. frugiperda*. Accordingly, nanoparticles of siRNAs would be a likely vector for any tropical application (Ana et al., 2020; Shuo et al., 2020); pheromone bait traps are a sprayable RNAi product (Baum and Roberts, 2014). The establishment of transgenic *S. frugiperda* expressing *Caenorhabditis elegans* systemic RNA interference defective protein 1 (*CeSid1*) provided possibilities for employing CeSid1 to enhance RNAi efficiency in insects that show variable and inefficient RNAi (Chen et al., 2021). Moreover, key genes associated with the development, detoxification, digestion and defense of the midgut have been identified as ideal targets for RNAi in *S. frugiperda* (Rodríguez-de la Noval et al., 2019), calling for future investigations to focus on these genes to enhance RNAi effectiveness. According to Rodríguez-dela Noval et al. (2019)and Tabashnik and Carrière (2017), the efficacy of RNAi could be significantly improved by pyramiding and expressing dsRNAs of these key genes targeted to the midgut of *S. frugiperda*. Likewise, the utilization of protein carriers with specific properties such as lectin and peptides, has been confirmed to provide protection for dsRNA and ensure RNAi efficiency (Parsons et al., 2018; DeSchutter et al., 2021). Knockdown of the expression of REase or dsRNases can enhance on RNAi efficiency and thus represents a target for further research of RNAi efficiency in *S. frugiperda* (Ruo-Bing et al., 2018; Peng et al., 2019). In addition, the study should focus on the appropriate identification of all possible causes of inefficient RNAi in *S. frugiperda* to enhance its efficiency for sustainable management of the pest.

Application of the RNAi approach in combination with novel biological options that have a different mode of action can provide effective management of *S. frugiperda* thereby improving the efficacy of RNAi-based technology (Griebler et al., 2008; Baum and Roberts, 2014; Ren et al., 2019). According to Jonathan (2015), RNAi is considered a plausible component of IPM in controlling *S. frugiperda.* Interestingly, push-pull technology (PPT) and botanical insecticides are among potential candidates to be deployed with RNAi in an integrated fashion. Currently, many studies have shown that the application of bioformulations of botanical materials (Jaoko et al., 2021) and the emission of chemical compounds by PPT (Khan et al., 2018; Midega et al., 2018; Mutyambai et al., 2019) provide promising results against *S. frugiperda* across diverse agro-ecologies, particularly in the African context. For instance, botanical extracts from *Melia volkensii* L, *Azadirachta indica* Juss, *Schinnus mol* L and *Phytolacca dodecandra* L. displayed antifeedant activity with larval mortality of more than 90% (Sisay et al., 2019; Jaoko et al., 2021). As a versatile option, PPT improves the aboveground and belowground plant environment to enhance the plant while making it less suitable for *S. frugiperda* (Khan et al., 2018; Sobhy et al., 2022) and possesses a convincing trait to act with RNAi. In other words, the importance of combining different control strategies is signified to improve the insecticidal activity of bio-options and prevent pest resistance emergence (Wang et al., 2017; Jaoko et al., 2021). Surprisingly, RNAi offers an exceptional mode of action among bioinsecticides through the mechanism of gene suppression and therefore can supplement the present methods used for pest control (Griebler et al., 2008; Tabashnik and Carrière, 2017; Mehlhorn et al., 2021). The deployment of RNAi with these candidate options does not enhance only enhances its efficiency but also provides a great contribution to ensuring the development of the IPM program. However, research evidence is lacking concerning the combined application and/or effect of these biological options to optimize their integration for better bioactivity against *S. frugiperda.* Hence, research emphasis is needed to determine the optimum combinations of RNAi with these options across diverse ecologies to boost their biological activity against the pest and verify their agronomic suitability.

In general, since endogenously generated and exogenously supplied dsRNAs activate RNAi in a sequence-specific manner, this strategy is a promising avenue for providing low-risk and environmentally safe plant protection (Dias et al., 2018; Hernández-Soto and Chacón-Cerdas, 2021). There is plenty of evidence of its use through host-induced gene silencing techniques to control insects (Shuo et al., 2020; Hernández-Soto and Chacón-Cerdas, 2021). No single protocol can be applied to every gene in every insect and the target tissue (Mehlhorn et al., 2021). Thus, the adaptability of RNAi methodology to control a particular pest must be carefully evaluated before deployment to maximize efficacy and sustainability (Davis-Vogel et al., 2018). The environmental risk assessment of RNAi-based genetically modified plants and new strategies, including direct spraying of dsRNA bioinsecticide should be particularly defined according to the objectives of each RNAi bioassay and experimental setup and are crucial for appropriate insect RNAi research as well as for commercial success (Baum and Roberts, 2014; Christiaens et al., 2018b; Liu et al., 2020; DeSchutter et al., 2021).

## Conclusion

RNAi-based technologies have tremendous potential as an effective and noninvasive approach for invasive insect pest management in modern agricultural systems. Most studies have reported promising achievements in RNAi application to control *S. frugiperda*. Despite increasing trends in RNAi application in *S. frugiperda*, it is not as straightforward as that observed for other insect species. Generally, lepidopterans, including *S. frugiperda*, are refractory to effective gene silencing by RNAi, especially when using oral delivery of dsRNA. Various factors could contribute to the efficiency of RNAi technologies to achieve effective management of insect pests. On the other hand, strategies such as the use of modified or concatenated dsRNAs have been used to improve the efficiency of RNAi in *S. frugiperda*. Moreover, the emergence of alternatives combining RNAi gene silencing with the induction of resistance in crops or multiple silencing is an opportunity to overcome the ongoing challenges in managing *S. frugiperda*, an invasive insect pest in agriculture. Hence, integration of this technology with any other compatible options is plausible in devising integrated pest management strategies. In addition, further research should be emphasized for a better understanding of RNAi-cellular interactions and insect gene silencing mechanisms as well as other influencing factors to enhance the efficiency of RNAi for the effective management of *S. frugiperda*. Further studies should also focus on the assessment of risk associated with RNAi-based insect pest management approaches.
